# AAV‐mediated Gene Therapy for Hereditary Deafness: Progress and Perspectives

**DOI:** 10.1002/advs.202402166

**Published:** 2024-11-18

**Authors:** Liyan Zhang, Fangzhi Tan, Jieyu Qi, Yicheng Lu, Xiaohan Wang, Xuehan Yang, Xiangyan Chen, Xinru Zhang, Jinyi Fan, Yinyi Zhou, Li Peng, Nianci Li, Lei Xu, Shiming Yang, Renjie Chai

**Affiliations:** ^1^ State Key Laboratory of Digital Medical Engineering Department of Otolaryngology Head and Neck Surgery Zhongda Hospital School of Life Sciences and Technology School of Medicine Advanced Institute for Life and Health Jiangsu Province High‐Tech Key Laboratory for Bio‐Medical Research Southeast University Nanjing 210096 China; ^2^ Co‐Innovation Center of Neuroregeneration Nantong University Nantong 226001 China; ^3^ State Key Laboratory of Hearing and Balance Science Department of Neurology Aerospace Center Hospital School of Life Science Beijing Institute of Technology Beijing 100081 China; ^4^ Otovia Therapeutics Inc. Suzhou 215101 China; ^5^ Department of Otolaryngology‐Head and Neck Surgery Shandong Provincial ENT Hospital Shandong University Jinan Shandong 250022 China; ^6^ Senior Department of Otolaryngology Head and Neck Surgery Chinese PLA General Hospital Chinese PLA Medical School Beijing 100853 China; ^7^ State Key Laboratory of Hearing and Balance Science Beijing 100853 China; ^8^ National Clinical Research Center for Otolaryngologic Diseases Beijing 100853 China; ^9^ Key Laboratory of Hearing Science Ministry of Education Beijing 100853 China; ^10^ Beijing Key Laboratory of Hearing Impairment Prevention and Treatment Beijing 100853 China; ^11^ University of Electronic Science and Technology of China Chengdu 610072 China; ^12^ Southeast University Shenzhen Research Institute Shenzhen 518063 China

**Keywords:** AAV, gene therapy, hereditary deafness

## Abstract

Hereditary deafness is the most prevalent sensory deficit disorder, with over 100 identified deafness‐related genes. Clinical treatment options are currently limited to external devices like hearing aids and cochlear implants. Gene therapy has shown promising results in various genetic disorders and has emerged as a potential treatment for hereditary deafness. It has successfully restored hearing function in >20 types of genetic deafness model mice and can almost completely cure patients with hereditary autosomal recessvie deafness 9 (DFNB9) caused by the OTOFERLIN (*OTOF*) mutation, thus serving as a translational paradigm for gene therapy for other forms of genetic deafness. However, due to the complexity of the inner ear structure, the diverse nature of deafness genes, and variations in transduction efficiency among different types of inner ear cells targeted by adeno‐associated virus (AAV), precision gene therapy approaches are required for different genetic forms of deafness. This review provides a comprehensive overview of gene therapy for hereditary deafness, including preclinical studies and recent research advancements in this field as well as challenges associated with AAV‐mediated gene therapy.

## Graphical abstract

Gene therapy has successfully restored hearing function in hereditary autosomal recessive deafness 9 (DFNB9) caused by the OTOFERLIN (*OTOF)* mutation, providing a translational paradigm for gene therapy in other forms of genetic deafness. In this work, a systematic overview of the current state of gene therapy for hereditary deafness, with particular emphasis on the advancements and challenges encountered in both basic and clinical research is summarized.

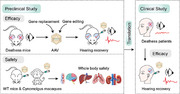

## 1 Introduction

Hearing loss can have a detrimental impact on an individual's language development, and quality of life, and can give rise to social and economic challenges. According to the World Health Organization, by 2050, at least 700 million people will require rehabilitation for hearing loss.^[^ ^1^ ^]^ Genetic factors account forward 50% of congenital hearing impairments,^[^ ^2^, ^3^ ^]^ and hereditary deafness manifests early in life and currently lacks specific pharmacological treatments. Cochlear implantation is the most commonly employed treatment, but the efficacy of cochlear implants is influenced by factors such as the integrity of the auditory nerve.

In 2012, researchers successfully restored hearing in mice by delivering the *Vglut3* gene^[^ ^4^ ^]^ into hair cells using recombinant adeno‐associated viruses (AAVs), marking an initial step toward gene therapy for deafness. Since then, significant progress has been made in gene therapy for hereditary deafness caused by mutations of genes such as including *Otof*, *Tmc1*, and *Pcdh15*, and some treatments have restored the hearing of mouse models to wild‐type levels.^[^ ^5^, ^6^, ^7^, ^8^, ^9^, ^10^, ^11^, ^12^, ^13^ ^]^ Notably, in clinical trials of gene therapy for treating autosomal recessive deafness 9 (DFNB9), the patients' hearing was recovered without evident adverse reactions.^[^ ^14^, ^15^, ^16^ ^]^ Nevertheless, numerous challenges persist in using gene therapy for treating deafness due to the intricate structure of the cochlea. These complexities amplify both therapeutic effectiveness requirements and demands on precision. The purpose of this study is to systematically summarize the preclinical and clinical research progress as well as the practical challenges of using gene therapy for hereditary deafness and thus provide perspective for the clinical application of gene therapy for hereditary deafness.

## 2 Physiologic Basis of Hereditary Deafness

### 2.1 The Complex Cochlear Structure

**Figure 1:**
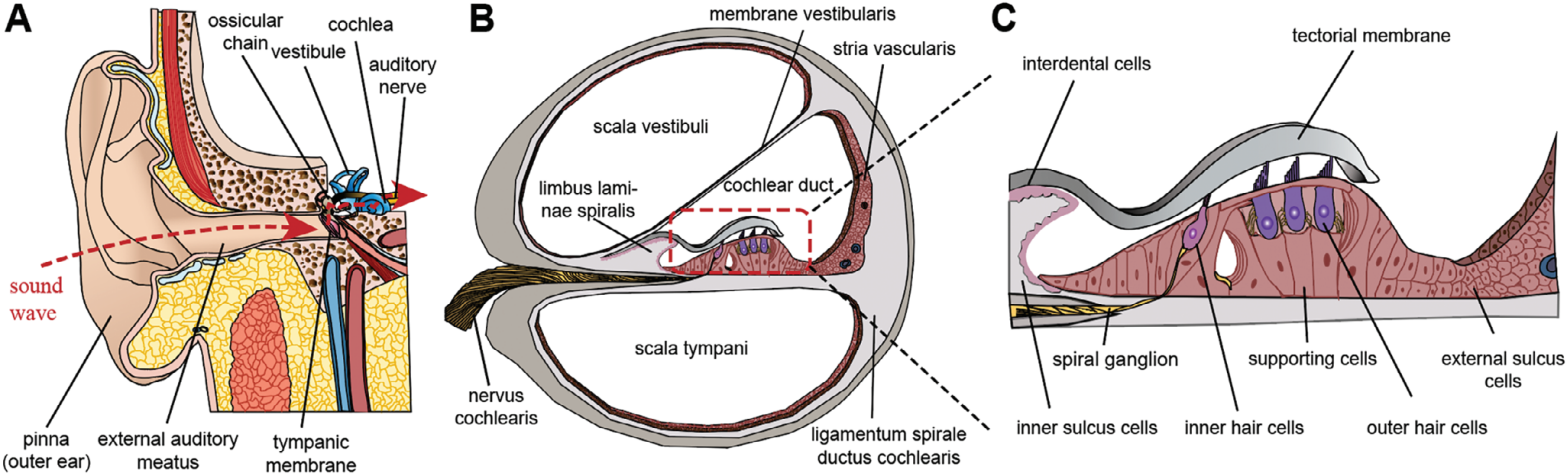
The structure of cochlea. A) Process of sound conduction in the ear. B) Anatomy of the cochlea. C) An enlarged diagram depicting the structure of the organ of Corti.

The human ear can be anatomically divided into the outer, middle, and inner ear (**Figure** **1**A). Sound waves from the environment travel through the external auditory canal to the tympanic membrane of the middle ear, which subsequently causes mechanical vibration of the auditory ossicle chain, converting the sound signals into electrical impulses in the inner ear. The electrical signals then travel through neurons to reach the auditory cortex and produce hearing.^[^ ^17^, ^18^ ^]^

The cochlea has a complex, snail‐shell‐like structure and plays an indispensable role in hearing and auditory transduction. The osseous cochlear duct comprises two cavities, namely the superior and inferior cavity. The superior cavity is further divided by the vestibular membrane into two compartments known as the scala vestibuli and cochlear duct, while the inferior cavity, scala tympani remains separate. Among these components, the organ of Corti resides above the basilar membrane and serves as a pivotal sound receptor during auditory transmission.^[^ ^19^ ^]^ The organ of Corti primarily consists of supporting cells, the tectorial membrane, hair cells, and the spiral ganglions (Figure 1B,C).

### 2.2 Classification of Hereditary Hearing Loss Genes

Sensorineural hearing loss (SNHL) is caused by abnormalities in the inner ear or auditory nerve, such as damage to hair cells, auditory neurons, and supporting cells. Mutations in genetic material are a primary contributor to sensorineural hearing loss. Currently, a total of 148 genes associated with hereditary deafness have been identified (https://hereditaryhearingloss.org/, updated on February 4, 2024). These genes are expressed in multiple cell types (**Table** **1**).

**Table 1:** Genes associated with hearing loss and their expression sites.^[^ ^20^ ^]^

Expression site	Gene symbol
Tectorial membrane	*Cldn14, Ceacam16, Col2a1, Col9a1, Col9a3, Col11a1, Otog, Otogl, Tecta*
Interdental cell	*Gjb2, Gjb6, Ceacam16, Edn3, Clic5, Wfs1, Otoa, Otog, Otogl, Chd7, Eya1, Sox10*
Spiral limbus	*Myh9, Gjb2, Gjb3, Gjb6, Esrrb, P2rx2, Wfs1, Col2a1, Col4a3, Col4a5, Col9a1, Col9a3, Col11a2, Otoa, Chd7, Eya1, Eya4, Serpinb6, Ccdc50*
Inner sulcus cell	*Myh9, Gjb2, Gjb6, Edn3, Wfs1, Col4a3, Col4a4, Col4a5, Eya1, Eya4, Sox10, Adcy1, Clpp, Serpinb6*
Spiral ganglion	*Edn3, Esrrb, P2rx2, Slc17a8, Wfs1, Col4a6, Chd7, Eya1, Eya4, Pax3, Sox10, Cabp2, Tbc1d24, Gipc3, Kars, Msrb3, Pnpt1, Prps1, Tmprss3, Clrn1, Dfnb59, Otof, Tbc1d24, Tspear, Mir96*
Inner hair cell	*Actg1, Espn, Rdx, Syne4, Triobp, Myh14, Myo3a, Myo6, Myo7a, Myo15a, Cdh23, Pcdh15, Cldn14, Marveld2, Tjp2,Ceacam16, Gpr98, Pdzd7, Tprn, Ush1c, Whrn, Edn3, Ednrb, Eps8, Esrrb, Ildr1, Vlgr1, Clic5, Lhfpl5, Loxhd1, P2rx2, Slc17a8, Tmc1, Wfs1, Otoa, Otogl, Strc, Chd7, Eya1, Pou4f3, Six1, Cabp2, Cib2, Gpsm2, Adcy1, Clpp, Gipc3, Grxcr1, Grxcr2, Kars, Lrtomt/Comt2, Msrb3, Pnpt1, Prps1, Ptprq, Serpinb6, Tmprss3, Clrn1, Dfnb59, Elmod3, Mir96, Otof, Ush2a, Sans, Smac/Diablo, Smpx,Tbc1d24, Tmie, Tspear*
Supporting cell	*Myh9, Myh14, Syne4, Triobp, Gjb2, Gjb6, Cldn14, Tjp2, Ceacam16, Tprn, Esrrb, Ildr1, Lhfpl5, P2rx2, Wfs1, Otogl, Eya1, Sox10, Cib2, Gpsm2, Adcy1, Clpp, Grxcr1, Kars, Lrtomt/Comt2, Msrb3, Pnpt1, Ccdc50, Elmod3, Smpx, Tmie*
Outer hair cell	*Actg1, Espn, Rdx, Syne4, Triobp, Myo3a, Myo6, Myo7a, Myh9, Myh14, Myo15a, Cdh23, Pcdh15, Cldn14, Marveld2, Tjp2, Ceacam16, Gpr98, Pdzd7, Tprn, Ush1c, Whrn, Edn3, Esrrb, Eps8, Ildr1, Vlgr1, Clic5, Kcnq4, Lhfpl5, Loxhd1, P2rx2, Slc26a5, Tmc1, Wfs1, Otogl, Strc, Chd7, Eya1, Pou4f3, Six1, Cabp2, Cib2, Gpsm2, Adcy1, Clpp, Gipc3, Grxcr1, Grxcr2, Kars, Lrtomt/Comt2, Msrb3, Pnpt1, Prps1, Ptprq, Serpinb6, Ccdc50, Clrn1, Dfnb59, Elmod3, Mir96, Otof, Sans, Smac/Diablo, Smpx, Tbc1d24, Tmie, Tspear, Ush2a*
External sulcus cell	*Myh9, Myh14, Gjb2, Gjb6, Slc26a4, Wfs1, Coch, Col4a3, Col4a4, Col4a5, Col11a1, Col11a2, Eya4, Sox10, Serpinb6*
Stria vascularis	*Myh14, Gjb2, Gjb6, Marveld2, Edn3, Esrrb, Kcne1, Kcnj10, Kcnq1, Wfs1, Col4a3, Col4a5, Col4a6, Col11a1, Col11a2, Chd7, Eya1, Grhl2, Pax3, Six1, Sox10, Serpinb6, Bdp1, Ccdc50, Ndp, Tmie, Tsper*
Spiral ligament	*Myh9, Myh14, Gjb2, Gjb3, Gjb6, Esrrb, Wfs1, Coch, Col4a3, Col4a4, Col4a5, Col4a6, Col9a1, Col9a3, Col11a1, Col11a2, Chd7, Eya1, Pou3f4, Crym, Serpinb6, Bdp1, Ccdc50*
Reissner's membrane	*Myh9, Cdh23, Cldn14, Tprn, Esrrb, P2rx2, Wfs1, Col4a3, Col4a5, Chd7, Eya1, Grhl2, Pou3f4, Sox10, Bdp1, Grhl2, Tmie*

### 2.3 Conventional Clinical Treatment Strategies for Hereditary Deafness

Currently, the primary therapeutic modalities for hereditary hearing loss are the use of hearing aids and cochlear implants. Hearing aids serve to amplify auditory stimuli in individuals with mild to moderate hearing impairment, whereas cochlear implants are suitable for patients exhibiting more profound degrees of auditory deficit.^[^ ^21^ ^]^ However, both hearing aids and cochlear implants are ineffective for patients with damage to the spiral ganglion neurons.^[^ ^22^ ^]^ Additionally, after implantation, cochlear implants may lead to residual hearing loss.^[^ ^23^ ^]^ Also, both cochlear implants and hearing aids are unable to improve pathological conditions in the inner ear. Moreover, due to the high variability of genetic variants associated with hearing loss, a diverse range of therapeutic strategies may be necessary. In response to these challenges, gene therapies such as gene replacement and gene editing to repair or restore the expression of specific genes have instilled renewed optimism in the treatment of hereditary deafness.^[^ ^24^, ^25^, ^26^, ^27^ ^]^

## 3 Basic Research on Gene Therapy for Hereditary Deafness

### 3.1 Overview of the Current Status of Gene Therapy for Hereditary Deafness

Gene therapy involves the delivery of exogenous genetic material (DNA or RNA) to target cells and the use of these nucleic acid sequences and their corresponding expression products for the regulation, repair, replacement, or deletion of pathogenic genes in these cells. These approaches seek to mitigate or restore the dysfunction caused by gene defects and hold significant potential for treating genetic deafness, as evidenced by recent successful reports of AAV‐mediated gene therapy strategies for restoring hearing function in patients with *OTOF* mutations.^[^ ^14^, ^15^, ^16^ ^]^

### 3.2 Gene Therapy Vectors for Deafness

Commonly employed viral vectors in gene therapy for deafness encompass adenovirus (AdV), AAV, lentivirus (LV), retrovirus (RV), etc., with AAV being the predominant vector. Detailed characteristics of these frequently used viral vectors are presented in **Table** **2**.

**Table 2:** Types and features of commonly used viral vectors.

Virus type	Common Serotypes	Capacity	Genome	Feature
AdV^[^ ^30^, ^31^, ^32^ ^]^	Ad3、 Ad5、 Ad26	7.5–36 kb Generation 1: up to 7.5 kb Generation 2: up to 14 kb Generation 3: up to 36 kb	dsDNA	Extrachromosomal, Long‐term expression, Broad tissue tropism, High immunogenicity
AAV^[^ ^29^, ^33^ ^]^	AAV1‐12	4.8kb	dsDNA	Extrachromosomal, Long‐term expression, Low immunogenicity
RV^[^ ^34^, ^35^ ^]^	MLV	8kb	ssRNA	Integration, Long‐term expression, Infect dividing cells
LV^[^ ^36^, ^37^ ^]^	HIV‐1	9–10kb	ssRNA	Integration, Long‐term expression, Infect dividing and non‐dividing cells

AAV is a non‐enveloped DNA virus with a single‐stranded genome that was initially discovered and isolated in 1965 from adenovirus preparations obtained from primates.^[^ ^28^ ^]^ Due to the indispensable role of adenovirus and other herpes viruses as helper viruses, it was designated as an adenovirus‐associated virus and categorized within the genus of dependent viruses.^[^ ^29^ ^]^

The widespread clinical application of AAV is attributed to its reduced immunogenicity and capsid toxicity, making it a preferred choice in medical practice. There have been 63 ongoing or completed clinical trials utilizing AAV‐based interventions (https://clinicaltrials.gov/, with the filters Intervention/treatment: adeno‐associated virus; Title / Acronym: AAV; Applied Filters: Interventional, updated on January 2024). Meanwhile, more than 30 AAV gene therapy drugs have been declared and approved in China (https://www.cde.org.cn/, updated on December 2023). There are currently 17 ongoing clinical trials utilizing AAV vectors (https://www.chictr.org.cn). Among these trials, Qi et al., Lv et al., and Wang et al. employed gene therapy to restore auditory function in individuals with congenital DFNB9 resulting from *OTOF* mutations (NCT05901480 and ChiCTR2200063181).^[^ ^14^, ^15^, ^16^ ^]^

### 3.3 Structure of AAV

AAV is composed of two distinct components, namely the outer capsid and the inner genome. AAV harbors a single‐stranded DNA molecule of 4.7 kb in length, which encompasses a *rep* gene and a *cap* gene flanked by an inverted terminal repeat (ITR). The external structure comprises an icosahedral capsid consisting of three capsid proteins (VP1, VP2, VP3), with dimensions ranging from 20 nm to 25 nm.^[^ ^38^ ^]^ Currently, there are at least 12 wild‐type AAV serotypes with over 100 variants, and novel AAV mutants are continuously being generated within these vectors to enhance gene delivery efficiency.^[^ ^39^ ^]^ Due to variations in vector affinity toward cell surface glycoprotein receptors, secondary receptors, or potentially the co‐receptor AAVR, each serotype demonstrates distinct tissue tropism.^[^ ^40^, ^41^, ^42^ ^]^ In recent years, AAV has emerged as the preferred vector for gene therapy regimens due to its exceptional safety profile, broad tissue and cellular tropism, and high infective efficiency.^[^ ^43^ ^]^

Recombination AAV (rAAV) is a recombinant variant derived from wild‐type and non‐pathogenic AAV.^[^ ^44^ ^]^ The native coding sequences, such as the *rep* gene and *cap* gene, as well as the non‐coding DNA sequences (promoter) between its ITR regions, are substituted with the transgene cassette. Target genes are inserted into the transgene cassette to achieve specific genetic modifications. This modification renders replication in the rAAV genome impossible due to the absence of the *rep* and *cap* genes, allowing only delivery of the gene of interest into target cells.^[^ ^45^ ^]^ The rAAV vectors possess numerous advantages including stable physicochemical properties, low pathogenicity, minimal integration risk, and sustained exogenous gene expression. Consequently, they have emerged as one of the most extensively investigated and applied vectors in vivo for gene therapy purposes.

### 3.4 The Capsid of AAV

Currently, gene therapy in the cochlea primarily focuses on targeting hair cells and supporting cells. Gene therapy involves repairing genes in situ and regenerating hair cells derived from supporting cells. AAV capsid species exhibit broad diversity, and they vary in their ability to target and transduce the inner ear efficiently. While most AAV serotypes can effectively infect inner hair cells, they show lower efficiency in infecting outer hair cells and supporting cells.^[^ ^46^, ^47^, ^48^ ^]^ Furthermore, the transduction rate of AAV vectors within the inner ear is influenced by the animal's age.^[^ ^49^ ^]^ **Table** **3** presents the commonly used AAV capsids along with their respective transduction rates for different cell types within the mouse inner ear.

**Table 3:** Transduction rates of commonly used AAV capsids to different cells of the inner ear.

Common AAV Serotypes	Stage	Hair Cell		Supporting Cell
		IHC	OHC	
AAV1^[^ ^46^, ^52^ ^]^	neonatal	+++++	++	−
	adult	++	∖	+
AAV2^[^ ^46^, ^52^, ^53^, ^54^ ^]^	neonatal	++++	+++	+
	adult	++++	++	+
AAV8^[^ ^55^ ^]^	neonatal	++++	++	+
	adult	++++	+	‐
AAV9^[^ ^46^, ^52^, ^56^ ^]^	neonatal	++++	+++	+
	adult	+++	∖	+
AAV‐S^[^ ^57^ ^]^	neonatal	+++++	++	+
	adult	+++++	++	−
AAV‐DJ^[^ ^58^, ^59^ ^]^	neonatal	+++	+	+++
AAV‐PHP.eB^[^ ^58^, ^60^ ^]^	neonatal	+++++	++++	∖
AAV2.7m8^[^ ^61^ ^]^	neonatal	++++	+++	+
	adult	++++	++++	−
Anc80L65^[^ ^62^, ^63^, ^64^, ^65^ ^]^	embryonic	++++	++++	+++
	neonatal	+++++	++++	−
	adult	+++++	+++	−
AAV‐ie^[^ ^65^ ^]^	neonatal	++++	+++	++++
	adult	++++	−	++++
AAV‐PHP.B^[^ ^66^ ^]^	neonatal	+++	++	−
	adult	++++	∖	−

“+” indicates the infection efficiency of each commonly used serotype against different cell types in mice of different ages.; “−” indicates that the experiments in the literature did not explicitly draw relevant conclusions.; “∖” indicates that the experiments in the literature resulted in almost no transduction or total death.; IHC: inner hair cell. OHC: the outer hair cell.

The AAV serotypes that are currently available are inadequate for targeting all cell types that express deafness‐related genes. For instance, *GJB2* mutations are the most prevalent genetic causes of non‐syndromic hearing loss in humans and are expressed in non‐sensory cochlear cells such as supporting cells, interdental cells, inner sulcus cells, the stria vascularis, and the spiral ligament.^[^ ^50^, ^51^ ^]^ Unfortunately, there are limited AAV vectors that efficiently and consistently target these supporting cells. Therefore, it is crucial to screen for AAV vectors with both enhanced tropism toward disease‐causing gene‐expressing cells and improved infective efficiency.

### 3.5 Basic Research on AAV‐mediated Hereditary Deafness Gene Therapy

**Figure 2:**
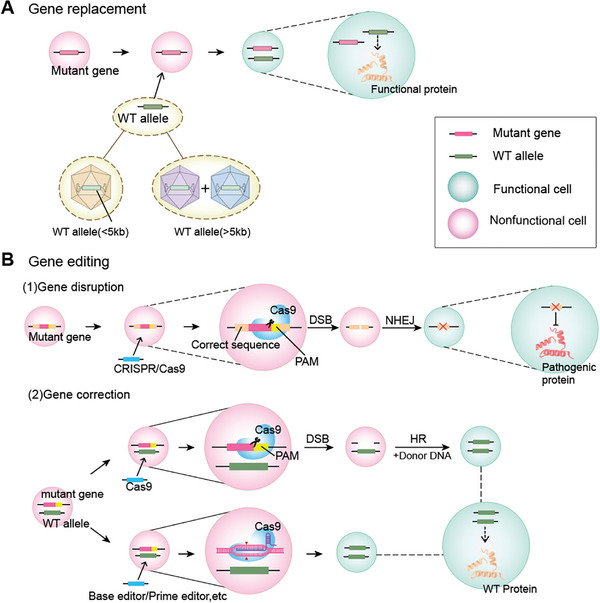
Gene therapy strategies for hereditary deafness. A) The gene replacement strategy. B) The gene editing strategy. DSB:double‐strand breaks, HR: homology‐directed repair, NHEJ: nonhomologous end joining, PAM: protospacer adjacent motif.

Gene therapy strategies for hereditary deafness primarily encompass gene replacement and gene editing tools (**Figure** **2**). Gene replacement involves introducing a functional target gene sequence to compensate for the loss of function of the mutant gene. This approach is suitable for treating minor invisible mutation diseases and dominant genetic disorders with insufficient haploinsufficiency, making it the most used commonly strategy in the clinical treatment of hereditary deafness. The earliest study on ear gene therapy commenced in 2012 and demonstrated partial restoration of hearing function in *Vglut3*‐deficient mice through AAV1‐mediated gene replacement therapy.^[^ ^4^ ^]^ Since then, significant advancements have been made in the field of gene therapy for AAV‐mediated genetic deafness, further validating its feasibility in various deafness mouse models such as *Otof* ^Δ/Δ^, *Tmc1* ^Y182C/Y182C^, *Tmc2* ^Δ/Δ^, *Strc* ^Δ/Δ^, and *Kcnq4* ^W276S/+^ (**Table** **4**).

**Table 4:** Summary of gene replacement for improving the hearing function in mouse models.^[^ ^25^ ^]^

Gene	Mouse Model	Injection Time	Delivery Route	Vector‐Promoter	Dosage	Expression Site
*Vglut3*	*Vglut3^−/−^*	P0‐P3, P10‐P12^[^ ^4^ ^]^	RWM, cochleostomy	AAV1‐AM/CBA	0.6–1 µL, 2.3 × 10^13^ vg mL^−1^	IHCs
		5, 8 and 20 weeks^[^ ^67^ ^]^	PSCC	AAV8‐CMV	1 µL, 2.04 × 10^13^ vg mL^−1^	
*Gjb6*	*Gjb6^−/^* ^−[^ ^68^ ^]^	E11.5	Otocysts	Plasmid	3 µg/µL,/	SL, SV, SLi, SCs
*Gjb2*	*P0^cre^‐Cx26^fl/fl^* ^[^ ^69^ ^]^	P0, P42	RWM	AAV1‐CMV	/, 8.6 × 10^11^ vg	SL, SV, SLi, SCs
*Kcnq1*	*Kcnq1^−/^* ^−[^ ^70^ ^]^	P0‐2	SM, RWM	AAV1‐CBA	0.5 µL, 5.2 × 10^12^–1.5 × 10^13^ vg mL^−1^	SV
*Pjvk*	*Pjvk^−/^* ^−[^ ^71^ ^]^	P3	RWM	AAV8‐CAG	2 µL, 1 × 10^13^ vg mL^−1^	HCs
*Tmc1*	*Tmc1^−/−^*, *Tmc1* ^Bth/+[^ ^48^ ^]^	P0‐2	RWM	AAV1‐CBA	1 µL, 1.8–2.4 × 10^13^ vg G mL^−1^	HCs
	*Tmc1^−/^* ^−[^ ^72^ ^]^	P1‐2	RWM	Anc80L65‐CMV	1 µL, 1.4 × 10^14^ vg mL^−1^	
	*Tmc1^−/−^*, *Tmc1* ^Baringo[^ ^73^ ^]^	P1, P7	Utricle	PHP.B‐CMV	1 µL, 5.46 × 10^10^vg,	
*Msrb3*	*Msrb3^−/^* ^−[^ ^74^ ^]^	E12.5	Otocytst	AAV1‐CMV	0.6–1 µL, 1.31 × 10^13^ vg mL^−1^	HCs
*Ush1c*	*Ush1c* c.216G>A^[^ ^75^ ^]^	P0‐1, P10‐12	RWM	Anc80 L65‐CMV	0.8–1 µL, 1.31 × 10^13^ vg mL^−1^	HCs
*Ush1g*	*Ush1g^−/^* ^−[^ ^76^ ^]^	P2.5	RWM	AAV8‐CAG	2 µL, 1.47 × 10^13^ vg mL^−1^	HCs
*Whirlin*	*Whrn* ^wi/wi[^ ^77^ ^]^	P4	PSCC	AAV8‐CMV	0.98 µL 1 × 10^13^ vg mL^−1^	HCs
*Clrn1*	*Clrn1^ko/ko^* ^−[^ ^78^ ^]^	P1‐3	RWM	AAV2/AAV8‐smSBA	2 µL, 8.6 × 10^12^ vg mL^−1^, 3.41 × 10^13^ vg mL^−1^	HCs
	Clrn1^fl/fl^ Myo15^Cer^, *Clrn1^ex4^* ^−^ *^/^* ^[^ ^79^ ^]^	P1‐3	RWM	AAV8‐CAG	2 µL, 6 × 10^12^ vg mL^−1^	
	*Clrn1^−/^* ^−[^ ^66^ ^]^	P0‐P1	RWM	PHP.B‐CBA	1‐1.2 µL, 7.4 × 10^10^ vg	
*Lhfpl5*	*Lhfpl5^−/^* ^−[^ ^80^ ^]^	P1‐2	RWM	Exosome‐AAV1‐CBA	1‐1.2 µL, 2.7 × 10^9^ vg	HCs
*Otof*	*Otof^−/−^*	P6‐7^[^ ^81^ ^]^	RWM	AAV2/6‐CMV	1, 1.2 × 10^10^ vg or 1.38 × 10^10^ vg	IHCs
		P10, 17, 30^[^ ^10^ ^]^		AAV2 quadY‐F‐CMV	2 µL, 6.3 × 10^12^vg mL^−1^ + 4.5 × 10^12^ vg mL^−1^	
		P0‐2, P30^[^ ^7^ ^]^		PHP.eB‐CAG	2 µL 2/0.5 × 10^10^ vg	
		P0‐2, P30^[^ ^82^ ^]^		PHP.eB‐CAG/Myo15	2 µL, 1 × 10^10^ vg	
		P0‐2, P30^[^ ^6^ ^]^		AAV1‐Myo15	/, 7.5 × 10^9^ vg, 1.5/3/6 × 10^10^ vg	
	OTOF^Q939*/Q939*^	P30^[^ ^5^ ^]^	PSCC	Anc80L65‐Myo15	1–2 µL, 2.8 × 10^10^ vg	
*Slc26a4*	Slc26a4^tm1Dontuh/tm1Dontuh^, *Slc26a4^−/^* ^−[^ ^83^ ^]^	E12.5	Otocyst	AAV2/1‐CMV	0.6–1 µL, 1.08 × 10^13^ vg mL^−1^	Outer sulcus
*Strc*	*Strc^−/^* ^−[^ ^84^ ^]^	P0‐1	Utricle	PHP.B‐CMV	1 µL,1.57 × 10^11^ vg mL^−1^ + 3.1 × 10^11^ vg mL^−1^	OHCs
*Kcne1*	*Kcne1^−/^* ^−[^ ^85^ ^]^	P0‐2	PSCC	AAV1‐CBA	Low dose: 0.5‐1 µL, high dose: 1.5–2 µL, 1.5 × 10^13^ vg mL^−1^	SV
*Syne4*	*Syne4^−/^* ^−[^ ^86^ ^]^	P0‐1.5	PSCC	PHP.B‐CMV	1 µL, 7.7 × 10^12^ vg mL^−1^	OHCs
*Pcdh15*	*Myo15^cre^‐Pcdh15^fl/fl[^* ^13^ *^]^*	P0‐1	RWM	PHP.B‐CMV	/, 5 × 10^10^ vg	HCs

SM: Scala media, RWM: round window membrane, PSCC: posterior semicircular canal, HC: hair cell, SL: spiral limbus, SV: stria vascularis, SLi: spiral ligament, IHC: inner hair cell. OHC: outer hair cell, VG: vector genomes.

Notably, recent clinical trials have demonstrated the successful curing of DFNB9 caused by mutations in the *OTOF* gene using AAV‐*OTOF*. Qi et al. showed that the AAV‐*OTOF* gene therapy was first administered to patients aged 5 and 8 years old, respectively, resulting in significant improvements in auditory brainstem response (ABR) and pure tone audiology in the world^[^ ^14^ ^]^ (Advanced Science, 8th 1, 2024). Subsequently, Lv et al. and Wang et al. demonstrated that AAV1*‐OTOF* gene therapy significantly reduced the mean ABR threshold in patients with *OTOF* deficiency by 40–57 dB at frequencies ranging from 0.5 to 4 kHz, thereby effectively ameliorating hearing function^[^ ^15^, ^16^ ^]^ (Lancet, 24th 1, 2024; Nature Medicine, 5th 6, 2024).

Gene editing therapy encompasses two approaches, namely gene knockout and gene repair,^[^ ^25^ ^]^ which involve the utilization of diverse gene editing tools to achieve targeted site‐specific insertion, deletion, or replacement of gene sequences. This enables the disruption, restoration, or activation of target gene expression. Gene knockout is particularly applicable for dominant inherited deafness caused by pathogenic mutated alleles or those that impede normal allelic function. For instance, in 2014, Gao et al. achieved successfully in vivo CRISPR/Cas9‐mediated gene editing of inner ear cells using the SpCas9‐gRNA ribonucleoprotein complex. Subsequently, in 2018, they successfully restored hearing function in *Tmc1* ^Bth/+^ deaf mice using SpCas9‐gRNA technology.^[^ ^87^ ^]^ Gene repair encompasses various techniques such as homologous recombination repair mediated by a base editor, a lead editor, and the Cas9 nuclease. The homologous recombination‐dependent repair pathway utilizes wild‐type DNA as a template to accurately correct the mutation site of the target gene back to its wild‐type form. Notably, both base editors and prime editors enable gene repair without inducing double‐strand breaks in the DNA. The research progress on gene editing in a mouse model of hereditary deafness is summarized in **Table** **5** below.

**Table 5:** Summary of gene editing to successfully improve hearing function in mouse models.^[^ ^25^ ^]^

Gene	Mouse Model	Injection Time	Delivery Route	Vector‐Promoter	Dosage	Expression Site
	Gene knockout					
*Tmc1*	*Tmc1* ^Bth/+^	P0‐2^[^ ^87^ ^]^	Cochleostomy	Lipofectamine 2000	0.3 uL, Cas9+sgRNA: 25µm	HCs
		P1^[^ ^88^ ^]^	Inner ear	Anc80L65‐CMV	1 µL, 4.8 × 10^14^vg mL^−1^	
		P1^[^ ^73^ ^]^	utricle	PHP.B‐CMV	1 µL, 5.4 × 10^14^vg mL^−1^ + 5.8 × 10^14^vg mL^−1^	
*Myo6*	*Myo6* ^C442Y/+[^ ^89^ ^]^	P0‐2	SM	PHP.eB‐EFS	0.5 µL, 3.73 × 10^13^vg mL^−1^	HCs
*Kcnq4*	*Kcnq4* ^W276S/+[^ ^90^ ^]^	P1‐3	Utricle, PSCC, RWM, SM	Anc80L65‐CMV	1 µL, 1 × 10^12^vg mL^−1^	OHCs
	*Kcnq4 ^G299D^* ^/+[^ ^91^ ^]^	P1‐2	SM	PHP.eB‐CMV	0.5 µL, 1.16 × 10^13^vg mL^−1^ + 2 × 10^13^vg mL^−1^	
*Pcdh15*	*Pcdh15* ^av‐3J[^ ^92^ ^]^	P0‐2	SM	AAV2/9‐CMV	0.6 µL, 1.5 × 10^14^vg mL^−1^	HCs
	Gene correction					
*Klhl18*	*Klhl18* ^F^ *^lowf^* ^[^ ^93^ ^]^	P1	Inner ear	PHP.eB‐EF1α	0.5 µL, 2 × 10^13^vg mL^−1^ + 1 × 10^14^vg mL^−1^	IHCs
*Tmc1*	*Tmc1* ^Baringo[^ ^94^ ^]^	P1	Inner ear	Anc80L65‐Cbh	1 µL, 6.11 × 10^12^vg mL^−1^ + 8.26 × 10^12^vg mL^−1^	HCs
*Myo6*	*Myo6* ^C442Y/+[^ ^95^ ^]^	P0‐2	SM	PHP.eB‐EFS	0.5 or 0.3584 µL, 1.43 × 10^10^vg	HCs
*Otof*	*Otof^Q829X/Q829X^* ^[^ ^8^ ^]^	P0‐3, P5‐7, P30	SM, RWM	AAV9‐CMV	0.5 µL, 1 µL, 1–1.5 µL, 1.25 × 10^10^vg, 1.25 × 10^10^vg, 9.21 × 10^10^vg	IHCs

RWM: round window membrane, SM: Scala media, PSCC: posterior semicircular canal, HCs: hair cells, IHCs: inner hair cells. OHCs: outer hair cells, VG: vector genomes.

Besides DNA editing, in 2023, Xue et al. employed the CRISPR/Cas13 RNA single‐base editing tool (emxABE) delivered via a single AAV carrier to achieve precise repair of mutant mRNA in humanized *Otof* ^c.2485C>T^ (*Otof^Q829X/Q829X^*) point mutant mice,^[^ ^8^ ^]^ effectively restoring hearing to nearly normal levels in DFNB9 model mice. Currently, this study is undergoing clinical trials (NCT06025032). These findings indicate that gene delivery and editing techniques hold significant potential for substantial restoration of hearing across diverse mouse models.

### 3.6 AAV Delivery Routes in Mice

Local surgical administration of gene therapy drugs in the inner ear has garnered significant research interest due to its potential for addressing inner ear diseases that are not effectively treated with systemic medication, primarily because of the blood‐labyrinth barrier's limitation.^[^ ^96^, ^97^ ^]^ Compared to conventional drug delivery routes, local surgical injection enables targeted delivery of corrected genetic material for sustained treatment, obviates the need for multiple doses at challenging‐to‐access sites, and provides a more stable single‐dose environment within the cochlea. Commonly employed methods of administration in mice include cochleostomy, RWM) injection, and semicircular canal injection (canalostomy).

Cochleostomy enables direct delivery of the virus vector to the middle ear via the temporal bone, resulting in a high transfection rate.^[^ ^98^ ^]^ Despite minimal injection trauma, there is still a risk of endolymphatic leakage at the injection site. Therefore, this approach carries a high risk of hearing loss due to potential damage to internal cochlear structures. Kawamoto et al.^[^ ^99^ ^]^ compared transduction efficiency and postoperative outcomes between cochleostomy and canalostomy in adult mice and found that both methods were efficient for transduction, but cochlear function was impaired after cochleostomy injection. Chien et al.^[^ ^98^ ^]^ also demonstrated that surgical intervention through the RWM resulted in less trauma compared to cochleostomy while maintaining comparable transduction efficiencies. This suggests that cochleostomy, as an invasive procedure, may potentially disrupt inner ear balance and cause additional mechanical damage in mice. Therefore, the ideal delivery method should consistently offer high transduction efficiency and minimal associated damage rates. Notably, RWM injection reduces hair cell damage when compared to cochleostomy injection. This route enables medication delivery directly into the tympanic cavity and provides superior preservation of hearing sensitivity compared to cochleostomy. Posterior auricular window membrane injection is minimally invasive, offering excellent auditory protection with accelerated recovery.^[^ ^100^ ^]^ It is commonly employed in neonatal mice and non‐human primates (NHPs), enabling delivery of carriers to the perilymph of the cochlear scala tympani. Akil et al. concluded that transfection via the RWM is superior and more uniform compared to cochleostomy, allowing extensive transfection across various cell types including hair cells and spiral ganglion neurons.^[^ ^100^ ^]^ Moreover, it was demonstrated that ABR thresholds remained unaffected. Therefore, RWM injection can be considered a highly promising alternative for clinical small molecule transfection. However, the effectiveness and efficiency of drug delivery through the RWM primarily rely on the drug's permeability across the RWM and its residence time on the membrane.^[^ ^101^ ^]^ Therefore, considering the permeability of drugs or reagents on the RWM is crucial when designing experiments. Although surgical intervention via the RWM can be considered minimally invasive in neonatal mice, mild hearing loss has been observed in adult mice following RWM injection.^[^ ^98^, ^100^ ^]^ Zhu et al.’s experiments demonstrated a strong correlation between middle ear effusion, which occurs in mice after round‐window surgery, and hearing impairment.^[^ ^102^ ^]^

In contrast to the aforementioned methods, viral gene therapy administered through the posterior semicircular canal (PSCC) injection effectively transduces hair cells in both the vestibular nerve and the cochlea.^[^ ^64^ ^]^ During PSCC injections, the therapeutic agent is typically delivered by creating a hole in the PSCC. Zhu's research team discovered that the duration of peripheral lymphoid leakage and injection rate significantly impacted postoperative hearing outcomes, leading them to modify the conventional surgical technique for PSCC procedures.^[^ ^103^ ^]^ Although the rate of hair cell transduction was lower in adult mice compared to neonatal mice at equivalent viral loads, efficient transduction of adult mouse inner and outer hair cells was achieved in a dose‐dependent manner. Moreover, the PSCC is anatomically easy to localize in rodents because it is located outside the tympanic cavity. This facilitates surgical localization and manipulation while minimizing the risk of inner ear damage during surgery.^[^ ^104^ ^]^

Furthermore, the cochlear aqueduct has received only limited attention as a potential route for vector delivery, despite its direct fluid connection between the intracranial cerebrospinal fluid and the cochlear perilymph. This approach necessitates skull access and a larger injection volume compared to direct cochlear injection. In January 2023, Ranum et al.^[^ ^105^ ^]^ demonstrated robust transduction of hair cells and various other cells throughout the spiral ligament and spiral limbus by cerebrospinal fluid using all three AAV serotypes (AAV1, AAV2, and AAV9) in NHPs. Similarly, Mathiesen et al.,^[^ ^106^ ^]^ reported that the cochlear aqueduct exhibits lymphatic‐like properties as a bony channel connecting the cerebrospinal fluid to the inner ear fluid. The restoration of hearing in adult mice can be achieved by injecting a viral construct into the cisterna magna through the cochlear aqueduct. However, it should be noted that this effect was only evaluated after a two‐week treatment period, and further investigation is required to determine its long‐term efficacy. In order to enhance the validity of these findings, future research will focus on utilizing NHP models. Moreover, these results suggest that gene therapy administered via cerebrospinal fluid holds promising potential for addressing hearing loss.

## 4 Pre‐clinical Study in NHP

### 4.1 AAV Capsid and Transduction Efficiency

Currently, AAV‐mediated gene delivery has demonstrated efficacy and feasibility in mouse models; however, further validation is required for clinical applications. In addition to ensuring the effective transduction of AAV capsid‐mediated genes in cochlear hair cells, it is also essential to evaluate the transduction efficiency of these capsids in NHP cochlear hair cells (**Table** **6**). Various AAV vectors such as AAV1, AAV8, and AAV9 have shown high transduction efficiency in cochlear inner hair cells but limited effectiveness in outer hair cells. Conversely, variants based on the AAV9 capsid exhibit promising research potential and significant therapeutic effects in Usher syndrome 3A mice and *Otoferlin* mutant mice. Representative examples like PHP.B, AAV‐S, and Anc80L65, among others, hold crucial research significance for gene delivery therapy targeting hereditary hearing loss. As research progresses on the transduction efficiency of different capsids specifically targeting NHP cochlear cells, the clinical application of AAV‐mediated gene therapy appears imminent.

**Table 6:** Different AAVs for Transducing NHP or Human Inner Ear Cells.

Treatment reagent	Research objective	Delivery route	Animal model	Virus volume and dosage	Transduction efficiency
AAV9‐PHP.B‐GFP	Verification of HC transduction	RWM	Macaque	10µL High‐dose: 3 × 10^11^VG, Low‐dose: 1 × 10^11^VG	High‐dose: ≈92% in IHC/OHCs, Low‐dose: No obvious transduction in HCs.^[^ ^66^ ^]^
	Preclinical testing of transgenic expression in NHP cochlea	RWM	Cynomolgus monkeys (*Macaca fascicularis*)	10/20 µL Low‐dose: 1 × 10^11^VG Middle‐dose: 2 × 10^11^VG High‐dose: 3.5/7 × 10^11^VG	High‐dose: Almost 100% transduction in IHCs /OHCs Middle‐dose: ≈50% in IHC, ≈65% in OHC, Low‐dose: Almost 0% in HCs.^[^ ^107^ ^]^
	Transduction verification of fetal and adult inner ear implants	Explant culture method	Adult human vestibular organs, fetal human inner ear	18 µL 3.5 × 10^12^VG	54‐62% and 42–80% eGFP^+^ HCs in adult ampulla and utricle, 22‐52% and 53–69% eGFP^+^ HCs in fetal cochlear and utricular samples.^[^ ^108^ ^]^
AAV‐S‐EGFP	Proof of efficient NHP inner ear expression vector	RWM	Cynomolgus monkeys (*Macaca fascicularis*)	20 µL High‐dose:5.8/4.7 × 10^11^VG Low‐dose: 8 × 10^10^VG	High‐dose: Almost all types of cochlear cells are completely transduced, including 100% of IHCs, and 30%100% of OHCs, Low‐dose: The transduction efficiency of all cells in the cochlea was greatly reduced.^[^ ^57^ ^]^
AAV1/Anc80L65 ‐eGFP	Efficiency verification of carrier delivery in NHPs	RWM	Rhesus macaque	30 µL 2.55 × 10^11^VG	Anc80L65:Transduced 90% of the IHCs in the apical turn of the cochlea, AAV1:A downward trend transduction from the apex to the base.^[^ ^109^ ^]^
AAV1‐GFP	Clinical validation of the efficacy and safety of AAV1 in NHPs	RWM	*Macaca fascicularis *	20 µL High‐dose: 2.5 × 10^11^VG, Low‐dose: 1.5 × 10^11^VG	High‐dose: 63.16‐93.75% in IHCs; 27.78‐49.58% in OHCs, Low‐dose: 61.54‐87.5% in IHCs; 24.24‐61.54% in OHCs.^[^ ^6^ ^]^
Anc80L65‐EGFP	HC specific transfection	RWM	Cynomolgus monkeys	20‐40 µL /	Anc80L65‐Cmv‐EGFP: HCs and other inner ear cells, Anc80L65‐Myo15‐EGFP: only HCs.^[^ ^5^ ^]^

VG:  vector genomes, HC: hair cell, IHC: inner hair cell, OHC: outer hair cell, RWM: round window membrane.

### 4.2 Delivery Method

The therapeutic efficacy and surgical damage caused by inner ear drugs are determined by the delivery method employed. With the advent of gene therapy in clinical settings, the target audience for drug delivery has gradually expanded from mice to NHPs. Although there are variations in cochlear structures among animal models, most gene therapy delivery surgeries involve two main processes‐ surgical exposure and drug injection. Notably, the anatomical structure of the inner ear in NHP models closely resembles that of humans. In NHP models, a commonly utilized surgical approach for drug delivery involves exposing the round window through the mastoid facial recess.^[^ ^107^, ^109^ ^]^ This approach shares similarities with cochlear implant surgery, enabling clinical doctors to quickly acquire proficiency in this technique. Furthermore, the gene therapy company Akouos achieves effective transduction of inner hair cells by injecting the Anc80L65 vector through the RWM after creating holes in the stapes floor.^[^ ^109^ ^]^

### 4.3 Biosafety Evaluation

NHPs exhibit similarities to humans in terms of immunity and metabolism, making them valuable models for assessing the toxicity and pharmacology of AAV vector systems. In the study by Zhang et al., AAV1‐EGFP was administered to the inner ear of NHPs for 4 weeks, resulting in widespread distribution throughout various organs without any apparent systemic acute toxicity.^[^ ^6^ ^]^ Qi et al. conducted preliminary safety validation of AAV‐*OTOF* gene therapy in NHPs and demonstrated that local administration did not impact normal hearing.^[^ ^5^ ^]^ Following local injection into the inner ear of NHPs at different doses, AAV distribution was observed in organs such as the brain and liver. Although no organic lesions were detected during the short observation period, long‐term risks remain undetermined. Moreover, following local injection of the virus into the inner ear of NHPs, a rapid increase in AAV‐neutralizing antibodies can be detected in the blood. This finding emphasizes the need to consider the impact of virus delivery on immune organs during safety investigations pertaining to local injections. Consequently, these results suggest that long‐term safety monitoring should not only focus on the auditory system but also on encompass vigilance toward potential changes occurring in other organ systems.

## 5 Clinical Study of Gene Therapy for Hereditary Deafness

### 5.1 Clinical Trials

Currently, gene therapy for deafness remains in the phase of clinical research. The initial clinical trial of gene therapy for deafness was conducted by Novartis Pharmaceuticals (NCT02132130) utilizing CGF166, a recombinant adenovirus 5 vector containing cDNA encoding a human dysregulated transcription factor (*HATH1*). However, this trial involving 22 participants did not yield significant improvements in hearing recovery.^[^ ^110^ ^]^

**Figure 3:**
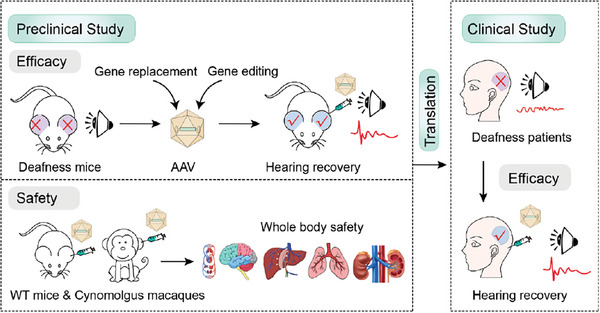
Preclinical research and clinical translation of DFNB9 gene therapy for hereditary deafness.

For hereditary deafness, there are currently 6 ongoing clinical trials worldwide, specifically focusing on DFNB9 (**Figure** **3**). These trials are being conducted by esteemed research teams including Otovia Therapeutics, Rrgener Therapeutics, HuidaGene, Akouos, Decibel Therapeutics, and Sensorion. Among these trials, five utilize AAV vector delivery to introduce full‐length *OTOF* DNA while one uses RNA editing.

Qi et al. utilized a hair cell‐specific promoter Myo15 to effectively deliver exogenous transgenes into the inner hair cells of mice and cynomolgus monkeys. AAV‐*OTOF* demonstrated stable and long‐lasting expression of full‐length *OTOF*, leading to hearing restoration in adult *Otof* point mutant mice for at least 150 days, reaching levels comparable to wild‐type mice. No evident systemic toxic reactions were observed in either mice or cynomolgus monkeys, with no discernible short‐ or long‐term effects on hearing, motor function, or memory.^[^ ^5^ ^]^ The results from two DFNB9 deafness patients (5‐year‐old participant (unilateral injection), 8‐year‐old participant (bilateral injection), NCT05901480) demonstrated a significant improvement in hearing function across all speech frequencies at 3 months, comparable to that observed in the contralateral ears with cochlear implants and approaching normal hearing levels^[^ ^14^ ^]^ (Advanced Science, 8th 1, 2024). Further long‐term observation is required to investigate potential additional effects on hearing in the gene therapy ear when using hearing aids and contralateral cochlear implants prior to gene therapy drug injections.

Similar findings were observed in Lv's and Wang's studies, which assessed the effectiveness and safety of AAV1‐*hOTOF* in 11 patients with *OTOF* mutations (ChiCTR2200063181). A single intra‐cochlear injection of AAV1‐*hOTOF* was administered through the RWM using an external auditory canal approach for 6 infants/children and bilateral ear gene therapy in 5 infants/children. During the 26‐week follow‐up period, none of the 11 patients experienced any toxic reactions or serious adverse events, and 10 patients exhibited recovery after treatment initiation, with a progressively more pronounced effect over time^[^ ^15^, ^16^ ^]^ (Lancet, 24th 1, 2024; Nature Medicine, 5th 6, 2024).

The gene therapy drug AK‐OTOF developed by Akouos received approval from the United States Food and Drug Administration (FDA) in September 2022 and is currently undergoing phase 1/2 clinical trials (NCT05821959). Following administration of a single dose of AK‐*OTOF* in the unilateral cochlea, the first patient exhibited complete hearing recovery across all tested frequencies within a span of 30 days. A threshold ranging from 65 to 20 dB HL was achieved, with some frequencies demonstrating hearing levels within the normal range. Both the surgical dosing procedure and AK‐*OTOF* therapy were well‐tolerated without any reported serious adverse events.^[^ ^111^ ^]^

Decibel Therapeutics used AAV1 and Myo15 to drive targeted expression of *OTOF*, resulting in sustained improvement of auditory function in mice for at least 52 weeks. The FDA granted approval for the DB‐OTO drug IND in October 2022. In the NCT05788536 trial, DB‐OTO was administered unilaterally into the cochlea. During the planned follow‐up visit, patients exhibited enhanced auditory response compared to baseline at week 6, as evaluated through ABR and behavioral pure‐tone audiometry.^[^ ^112^ ^]^

Sensorion employed dual‐AAV6 vectors to efficiently deliver the complete *Otof* sequence, resulting in transduction rates of up to 50% in inner hair cells and achieving hearing thresholds ranging from 40 dB to 60 dB in treated mice.^[^ ^10^ ^]^ Clinical trials investigating the therapeutic potential of OTOF‐GT (SENS‐501) have been initiated in France, Italy, and Germany; however, patient enrollment has not commenced.

Using the CRISPR/Cas13 RNA single‐base editing tool (emxABE), researchers successfully restored auditory function in homozygous *Otof^Q829X/Q829X^* point mutant mice, approaching levels comparable to those observed in wild‐type animals.^[^ ^8^ ^]^ The safety and tolerability of single round window inner ear administration in children with congenital deafness carrying the *Otof^Q829X/Q829X^* mutation is currently being investigated in a registered clinical trial at Clinicaltrials.gov (NCT06025032) but with no patient recruitment conducted thus far. These findings underscore the efficacy of gene therapy as a treatment approach for DFNB9 hereditary deafness, offering hope for gene therapy in other individuals affected by other types of hereditary hearing loss.

### 5.2 Safety Evaluation of AAV‐mediated Gene Therapy

Safety is the paramount concern in gene therapy clinical trials. The safety assessment of inner ear gene therapy drugs primarily aims to validate the reliability of drug delivery routes and ensure the safety of these therapeutic agents. Researchers opted for exposing the RWM during cochlear implantation surgery as a means of delivering gene therapy. After three months post‐surgery, cynomolgus monkeys exhibited no significant hearing loss, and there were no observed morphological or pathological changes in systemic organs, indicating that AAV delivery could be deemed safe.^[^ ^5^ ^]^

The immunogenicity and tissue tropism of AAV viruses have been extensively investigated. However, due to the widespread prevalence of AAV serotypes in the human population, the positive rate varies between 30% and 70%, depending on different serotypes and experimental cohorts.^[^ ^2^, ^113^, ^114^ ^]^ Therefore, the safety of AAV utilization remains a significant concern. The clinical trial conducted by Lv et al. excluded patients who developed AAV1‐neutralizing antibodies at titers exceeding 1:2000. All participants exhibited an increase in AAV1‐neutralizing antibodies and a negative T‐cell response to the AAV1 capsid from baseline to weeks 6 and 13.^[^ ^15^ ^]^ The presence of elevated concentrations of neutralizing antibodies in non‐responsive patients during clinical trials may contribute to the limited therapeutic efficacy that is observed.

The non‐pathogenic nature of AAV has traditionally been widely accepted; however, recent experiments and reports have presented challenges to this prevailing view. Notably, the study conducted by Gates et al. revealed a temporal association between AAV2 and unexplained cases of hepatitis.^[^ ^115^ ^]^ In August 2022, Novartis confirmed the occurrence of two fatalities in Russia and Kazakhstan ward 5 to 6 weeks after Zolgensma treatment in pediatric patients. Both cases were attributed to acute liver failure, a known adverse effect associated with Zolgensma administration. The higher dosage administered may have been the primary factor contributing to their morbidity. Subsequently, an additional report emerged from Italy where a patient treated with Zolgensma exhibited hemophagocytic lymphohistiocytosis, indicating excessive immune activation following gene therapy for spinal muscular atrophy. These incidents underscore the critical need for the comprehensive validation and consideration of AAV safety concerns. While no untoward reactions have been observed thus far in inner ear gene therapy, it is important to note that the observation period remains limited and that the long‐term follow‐up of patients is imperative.

### 5.3 Perspectives on Clinical Trials for Deafness

The progress in gene therapy for deafness has been remarkable, with *OTOF* serving as the earliest successful case and marking a great breakthrough, and this is poised to catalyze significant advancements in this field. Currently, research on gene therapy for hereditary deafness still primarily centers around the *OTOF* gene. This choice may stem from the fact that defects in the *OTOF* gene have relatively minimal impact on the inner ear's structure and environment, thereby preserving hair cell integrity to a large extent. Following the restoration of the *OTOF* gene, both hair cells and auditory circuits continue to exhibit normal functionality. These findings provide a foundation for treating patients with mutations in the *OTOF* gene.

Despite being the earliest genetic deafness successfully to be treated with gene therapy, hereditary DFNB9 still poses several questions regarding the application of such therapies. The efficacy and dosage of gene therapy for DFNB9 have not been thoroughly investigated, nor have long‐term safety tests been conducted. It is crucial to monitor patients over an extended period to determine if unilateral injection of the gene therapy drug affects hearing function on the contralateral side or if it has any impact on other physiological functions through the circulation and into the cerebrospinal fluid. Additionally, further research is needed to understand how different mutation types in *OTOF* may influence the drug's effectiveness in patients. Variations in hearing recovery among DFNB9 patients and their correlation with age at surgery raise questions about an optimal therapeutic window for gene therapy in this population. Lastly, because DFNB9 falls under the umbrella of auditory neuropathy spectrum disorders, it remains unknown whether gene therapy can restore speech recognition and hearing function specifically under noisy conditions.

DFNB9 is a rare disease that warrants attention toward common inherited deafness genes. *GJB2* mutations account for ward50% of autosomal recessive mutations and are among the most prevalent non‐syndromic deafness mutations. The Cx26 protein, encoded by the *GJB2* gene, is primarily expressed in supporting cells, stria vascularis, and spiral limbus.^[^ ^116^ ^]^ In a preclinical treatment study of *GJB2*, Otonomy reported that the drug OTO‐825 demonstrated efficacy in rescuing hearing loss in two animal models with a single dose. Sensorion and Akouos are also currently investigating *GJB2* gene therapy drugs, which are still in the early stages of basic research. Decibel Therapeutics is advancing AAV.103* drugs targeting *GJB2* mutations through the IND‐ENABLING process. However, the treatment strategies employed by these research teams and the efficacy of these drugs used on their patients remain unknown. Numerous clinical studies have demonstrated that nonsyndromic deafness caused by mutations in the *GJB2* gene exhibits diverse clinical phenotypes such as bilateral symmetry, age at onset, degree of deafness, and stability.^[^ ^117^ ^]^ Therefore, individuals with different mutations within the *GJB2* may still require personalized precision gene therapy.

## 6 Summary and Future Prospects

The development of gene therapy programs is predicated upon a comprehensive comprehension of the underlying mechanisms driving disease pathogenesis. DFNB9 is a form of hereditary deafness characterized by a well‐defined mechanism in which mutations in the *OTOF* gene result in auditory nerve conduction disorder, ultimately leading to deafness. Due to its clear pathogenesis, DFNB9 stands as the sole hereditary deafness disease for which hearing has been successfully restored through the clinical application of gene therapy drugs.

However, for common hereditary deafness genes such as *GJB2* and *SLC26A4*, the mechanisms underlying deafness are intricate and lack an efficient mouse model capable of accurately simulating disease onset. Patients with GJB2‐associated deafness retain at least some auditory hair cells and neurons, and their hearing loss is usually in the moderate to profound range. In contrast, Gjb2‐CKO/KO mice exhibit a large number of missing hair cells and neurons and rapidly progress to severe deafness, which does not mimic the clinical phenotype well.^[^ ^117^ ^]^ Currently, only the GJB2 R75W mutation,^[^ ^118^ ^]^ and the GJB2 35delG^[^ ^51^ ^]^ mutant mouse model established by Li et al. using the tetraploid embryo compensation technique mimic human deafness relatively well. However, there are no particularly good model mice for other mutation types. The mutant *SLC26A4* phenotype is characterized by inner ear malformations. Seven mouse models have been reported, none of which observed goiter. Three of these mouse models (Slc26a4^2Dontuh/2Dontuh^, Slc26a4^p.C565Y^, and Slc26a4^p. T721M^)^[^ ^119^, ^120^, ^121^ ^]^ have the normal hearing function, and the other four (Slc26a4^1Dontuh/1Dontuh^, Slc26a4^p.L236P^, Slc26a4^loop^, and Pds‐/‐)^[^ ^122^, ^123^, ^124^, ^125^, ^126^, ^127^ ^]^ have moderate to severe hearing function, abnormal vestibular function, etc. Therefore, achieving transformation for them remains a formidable challenge.

Furthermore, the diverse array of expressed deafness genes across different inner ear cells presents an additional hurdle for precise gene therapy, underscoring the criticality of identifying viral vectors that can accurately target these specific cells. Subsequently, a suitable mouse model was employed to investigate both the efficacy and safety of the gene therapy drug, followed by further assessment of its biosafety in NHPs. In clinical practice, it is essential to establish a more robust evaluation system for gene therapy, improve patient screening mechanisms and drug efficacy assessment criteria, optimize the surgical pathway for inner ear delivery, and provide standardized postoperative care. Furthermore, long‐term patient follow‐up is crucial to ensure drug safety within the patient's body.

AAV‐based gene therapy represents a promising therapeutic approach for hereditary deafness. This comprehensive review provides a systematic overview of the current state of gene therapy for hereditary deafness, with particular emphasis on the advancements and challenges encountered in both basic and clinical research.

## Conflict of Interest

The authors declare that they have no conflict of interest.
